# A Description of *Echinochasmus pseudobeleocephalus* n. sp. (Echinochasmidae) Based on Morphological and Molecular Data

**DOI:** 10.3390/ani13203236

**Published:** 2023-10-17

**Authors:** Kristina Andreevna Kalinina, Vladimir Vladimirovich Besprozvannykh, Yulia Viktorovna Tatonova, Mikhail Yurievich Shchelkanov

**Affiliations:** 1 Federal Scientific Center of the East Asia Terrestrial Biodiversity, Far Eastern Branch, Russian Academy of Sciences, pr-t 100-letiya Vladivostoka 159a, Vladivostok 690022, Russia; besproz@biosoil.ru (V.V.B.); ytatonova@gmail.com (Y.V.T.); adorob@mail.ru (M.Y.S.); 2 G.P. Somov Research Institute of Epidemiology and Microbiology, Russian Federal Service for Surveillance on Consumer Rights Protection and Human Wellbeing, Selskaya St. 1, Vladivostok 690022, Russia

**Keywords:** Echinochasmidae, *Echinochasmus*, morphological criteria, 28S rRNA, taxonomy, Russian Far East

## Abstract

**Simple Summary:**

Far Eastern trematodes of the genus *Echinochasmus* were studied. As the analysis of the nuclear 28S rRNA gene sequence showed, the examined Far Eastern individuals did not belong to the species *E. beleocephalus* despite their morphological similarities and represented a new species, *Echinochasmus pseudobeleocephalus* n. sp. An analysis of phylogenetic relationships in Echinochasmidae supported their status as an independent species. The subdivision of individuals of the genus *Echinochasmus* into two groups was also confirmed on the basis of the number of head-collar spines and the tail length in cercariae.

**Abstract:**

Adult individuals of *Echinochasmus pseudobeleocephalus* n. sp. were obtained during an experimental study on trematodes’ life cycle. An analysis of the morphometric characteristics of the developmental stages and involvement of first intermediate hosts, snails of the genus *Boreoelona*, in their life cycle, revealed the identity of the obtained trematodes to the European species *Echinochasmus beleocephalus* previously discovered in the south of the Russian Far East. However, an analysis of molecular data, in particular sequences of the 28S rRNA gene, showed that the Far Eastern trematodes examined do not belong to European *E. beleocephalus* despite their morphological similarities. An analysis of phylogenetic relationships within the family Echinochasmidae supported the status of *E. pseudobeleocephalus* n. sp. as an independent species. Our new data confirmed that the individuals attributed to *Echinochasmus* can be subdivided into two groups on the basis of the number of head-collar spines and the tail length in cercariae on an intergeneric level.

## 1. Introduction

The trematode family Echinochasmidae Odhner 1910 comprises numerous species that in their mature stage parasitize mammals including humans, birds, and, less commonly, reptiles [1,2]. It is also known that members of this family use only prosobranch snails as first intermediate hosts and mostly fish as second intermediate hosts. In some cases, in addition to fish, mollusks and tadpoles can be involved in their life cycle as second intermediate hosts [3,4].

On both specific and higher levels, the taxonomy of Echinochasmidae is mainly based on the morphological characters of mature individuals. Molecular data have been obtained for a relatively small number of species in this family. However, these data allowed Tkach et al. (2016) [5] to place the species combined in the subfamily Echinochasminae Odhner, 1910 into a separate family, Echinochasmidae Odhner, 1910 [2]. In addition, differences in molecular characteristics were found between individuals of *Echinochasmus* Dietz, 1909 on the generic level. Some of them had 24 head-collar spines and cercariae with a tail length comparable to the body length, while in others cercariae had a longer tail and adult worms had 20–22 spines. The latter clustered with species of the genus *Stephanoprora* Odhner 1902 [5,6,7].

During a parasitological study of freshwater prosobranch snails of the family Bithyniidae Gray, 1857, collected in a lake of the Arsenyevka River basin (Primorsky Krai, Russia), we found snails emitting cercariae that were morphologically similar to cercariae of the family Echinochasmidae. The subsequent experimental completion of their life cycles, a study of their developmental stages, and an analysis of molecular markers have shown that the trematodes belong to a species of the genus *Echinochasmus*. The results of the study are presented below.

## 2. Materials and Methods

### 2.1. Life Cycle and Morphology of Adult Individuals

One snail *Boreoelona ussuriensis* (Ehrmann in Büttner and Ehrmann, 1927) that emitted cercariae similar in morphological parameters to short-tailed cercariae of Echinochasmidae was found among the examined prosobranch snails of the family Bithyniidae collected in a lake of the Arsenyevka River basin. A map of sampling location is provided in the Supplementary Material. According to the available information about the life cycles of Echinochasmidae in the south of the Russian Far East [4], tadpoles are one of the second intermediate hosts for echinochasmids with short-tailed cercariae. To obtain metacercariae from emitted cercariae, tadpoles of *Rana dybowskii* Günther, 1876 caught in an artificial pond were used. First, 50 tadpoles from this pond were dissected to confirm the absence of trematode metacercariae. Three tadpoles were placed in a Petri dish with cercariae being emitted from the snail into the water. One day later, encysted cercariae were found in tadpoles’ visceral tissues. After that, the snail emitting cercariae was placed with 10 tadpoles in a container filled with 500 mL water. After a 4-h exposure to cercariae, the tadpoles were transferred into another container. On day 24 post-exposure, one of the infected tadpoles was dissected, and 12 metacercariae were obtained from its visceral tissue, which were morphologically similar to those of the genus *Echinochasmus*. Other tadpoles were fed to a duckling and a chicken: four tadpoles to each bird. After 8 days, 23 mature flukes were found in the small intestine of the duckling, while there were no flukes in the chicken.

The obtained adult individuals were washed, fixed in 70% ethanol, and then some of them were transferred to 96% ethanol for further DNA isolation. Whole mounts of the adult flukes were prepared by staining with carmine alum, dehydrating in a graded ethanol series, clearing in clove oil, and embedding in Canada balsam. All measurements are in micrometers (µm).

### 2.2. DNA Extraction, Amplification, and Sequencing

In the genetic analysis, one cercaria and two adult specimens representing the genus *Echinochasmus* were used. The adult specimens were obtained from cercariae through the experimental completion of the life cycle. DNA was extracted by the HotSHOT method [8]. Partial sequences of the 28S rRNA gene (*28S*) were amplified using the specific primers Digl2 (5′-AAG-CAT-ATC-ACT-AAG-CGG-3′, forward) and 1500R (5′-GCT-ATC-CTG-AGG-GAA-ACT-TCG-3′, reverse) [9]. For sequencing, internal primers were used: 900F (5′-CCG-TCT-TGA-AAC-ACG-GAC-CAA-G-3′, forward) [10] and 1200R (5′-GAA-GGA-CGA-ATC-GCT-TCG-TG-3′, reverse) [11]. To amplify the ITS2 spacer region, the following primers were used: forward 1/F [12] and reverse BD2 (5′-ATC-TAG-ACC-GGA-CTA-GGC-TGT-G-3′) [13]. The ITS2 spacer region was sequenced using external primers.

The resulting nucleotide sequences were visually checked using the FinchTV ver. 1.4.0 and aligned manually in MEGA ver. 5.03 [14]. Sequences of other members of the family Echinochasmidae were accessed from GenBank (Table 1). 

The length of the sequences used in the phylogenetic analysis was 731 bp taking into account alignment. Phylogenetic relationships were reconstructed using the Bayesian Inference (BI) algorithm in the MrBayes (BI) program [20] and the Maximum Likelihood (ML) algorithm in the PhyML 3.1 program. The optimal model GTR + I + G based on the Akaike information criterion was obtained in the jModeltest 2.1.5 program [21]. For the former (BI) phylogenetic reconstruction, 500,000 generations were performed; in the ML analysis, 100 repetitions were used. Both phylogenetic reconstructions had a similar topology, and, therefore, a consensus tree is presented here. Genetic distances (*p*-distances) between individual sequences and clusters were calculated using MEGA ver. 5.03.

## 3. Results

*Echinochasmus pseudobeleocephalus* n. sp.

Syn. *Echinochasmus beleocephalus* [4].

Host: *Anas platyrhynchos* dom. (experimental host).

Localization: small intestine.

Intensity of infection: 23 specimens.

First intermediate host: *Boreoelona ussuriensis*.

Second intermediate host: tadpoles of *Rana dybowskii* (experimental host).

Localization: visceral tissue.

Type locality: lake in the Arsenyevka River basin, south of the Russian Far East (44°44′ N, 133°57′ E).

Type-deposition: holotype No. 222-Tr; paratype Nos. 223-228-Tr.

This material was deposited in the parasitological collection of the Zoological Museum (Federal Scientific Center of the East Asia Terrestrial Biodiversity, Far Eastern Branch, Russian Academy of Sciences, Vladivostok, Russia) on 22 November 2022; e-mail: petrova@biosoil.ru.

Etymology: The species epithet indicates a coincidental visual similarity to *Echinochasmus beleocephalus*.

**Figure 1:**
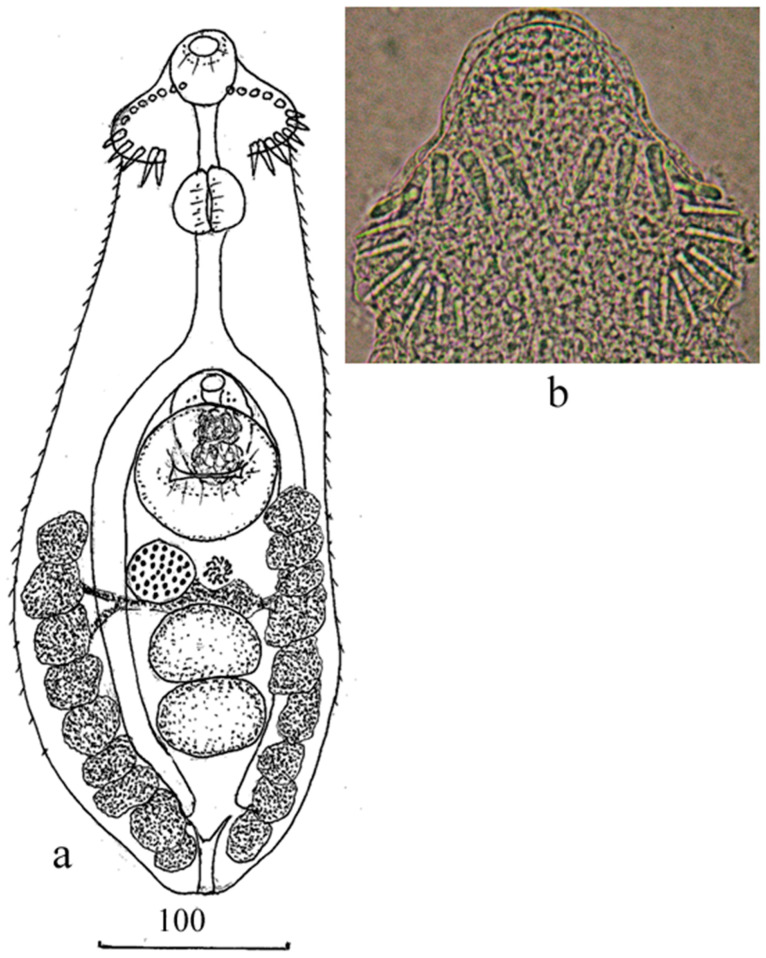
*Echinochasmus pseudobeleocephalus* n. sp. (**a**). Adult worm, (**b**). Head-collar.

Adult worm (based on seven specimens; Figure 1; Table 2). The body was elongated, covered by spines from the anterior end to the level of the posterior testis, with the most densely concentrated spines in the anterior third of the body. Oral sucker subterminal. Head-collar with 24 spines, arranged into single row interrupted dorsally. Prepharynx long; pharynx oval or rounded; esophagus longer than prepharynx. Intestinal bifurcation immediately anteriorly of cirrus-sac. Intestinal branches terminate, slightly separated from the posterior end of the body. Ventral sucker oval or rounded, in the middle third of the body. Testes two, tandem or slightly oblique, transversely-oval, adjacent to each other, in the posterior third of the body. Cirrus-sac oval, at median line of body and partly covered by ventral sucker. Internal seminal vesicle bipartite. Genital pore between intestinal bifurcation and anterior margin of ventral sucker. The ovary was rounded or transversely oval, sinistrally to the median line of the body, adjacent to the anterior testis, or at some distance anteriorly of the testis. Uterine seminal receptacle present. Mehlis’ gland left to the ovary, between the ventral sucker and anterior testis. Uterus short, located between the caeca, posterior margin of ventral sucker and anterior margin of the anterior testis, usually containing one or two large eggs. Vitelline fields lateral, extending from the level of the middle of the ventral sucker to the posterior end of the body. The vitelline reservoir on the median line of the body at the level of the anterior end of the anterior testis. Excretory vesicle Y-shaped. Stem of excretory vesicle short.

Molecular data. The nucleotide sequences of *28S* with a length of 1020 bp were identical between the three *Echinochasmus* specimens (one cercaria and two adult specimens).

The complete nucleotide sequences of the ITS2 region with a length of 695 bp were obtained. The resulting sequences were identical to that of the species *Echinochasmus japonicus* (MT268119). Thus, further analysis for this marker was not performed.

## 4. Remark

### 4.1. Morphological Identification

In their morphological characteristics, the experimentally obtained adult trematodes were similar both to *Echinochasmus japonicus* Tanabe, 1926 and to *E. beleocephalus* (Linstow, 1873). The former species was first discovered in Japan; subsequently, individuals identified as *E. japonicus* were found in Western Siberia, in the south of the Russian Far East, and in Vietnam [3,4,15,24]. Data on the life cycle and morphology of developmental stages were obtained for trematodes from each of the above-listed regions. The specimens of *E. japonicus* found in these regions differed slightly from each other in morphology, both at the stage of cercariae and at the adult stage. At the former stage, the main morphological differences between them were the presence of cuticular formations on the oral and ventral suckers in the specimens from Vietnam and Western Siberia and the absence of these formations in the Japanese and Russian Far Eastern specimens [3,4,15,25]. At the adult stage, there were differences in the number of head-collar spines: the Far Eastern individuals had 22 spines vs. 24 spines in individuals from other regions. The first intermediate hosts for Japanese, Vietnamese, and Russian Far Eastern trematodes were snails of the genus *Parafossarulus*; for West Siberian trematodes, snails of the genus *Codiella* Locard, 1894, also belonging to Bithyniidae.

Another representative of *Echinochasmus* from the Far Eastern region of Russia was attributed to the European species *E. beleocephalus*. It was identified on the basis of the life cycle and morphology of developmental stages [4,26,27]. However, these trematodes were also identical to the *E. japonicus* specimens from Vietnam and Western Siberia in the morphology of cercariae and adult individuals and had similarities with *E. japonicus* from Japan [24] and the Far East of Russia. They differed from the Japanese *E. japonicus* by the presence of cuticular formations on the suckers of cercariae and had a different number of spines on the head collar compared to those in *E. japonicus* from the Russian Far East (24 vs. 22, respectively).

As for the metric characteristics, adult trematodes identified as *E. japonicus* and *E. beleocephalus* had significant differences in the sizes of the body, oral and ventral suckers, pharynx, and cirrus sac (Table 2). For example, the *E. japonicus* individuals from Japan had lower values of most of these parameters than *E. japonicus* from the Russian Far East; the Vietnamese individuals of this species had smaller sizes of body length and cirrus sacs than the trematodes from both Japan and Russia. These sizes in the European *E. beleocephalus* were larger than in the Russian Far Eastern *E. beleocephalus*. However, in most of the parameters, the former individuals had the strongest metric similarity with *E. japonicus* from the Russian Far East (and differed only in the number of head-collar spines, as mentioned above), and the latter were similar to the Vietnamese *E. japonicus* (Table 2).

On the basis of the morphological characteristics of the developmental stages, the metric characteristics of adult trematodes (Table 2), and the features of their life cycles, the individuals obtained in the present study were identical to the Far Eastern flukes previously referred by Besprozvannykh (2009) [4] to as *E. beleocephalus*. Based on the above-listed morphological characteristics, the trematodes in our material, as well as those from the European and East Asia regions identified as *E. japonicus* and *E. beleocephalus*, are most probably representatives of a group of cryptic species. Among them, the individuals from the Far East of Russia identified as *E. japonicus* and having 22 head-collar spines (unlike other *E. japonicus* individuals), as well as *E. beleocephalus* individuals with 24 spines, most likely represented a separate species. For the definitive determination of species affiliation of the East Asian trematodes under study, molecular data for *E. japonicus* from the type locality are required. However, to date, nucleotide sequences have been obtained only for the specimens identified as *E. japonicus* from Vietnam and for those identified as *E. beleocephalus* from Europe.

### 4.2. Molecular Identification

**Figure 2:**
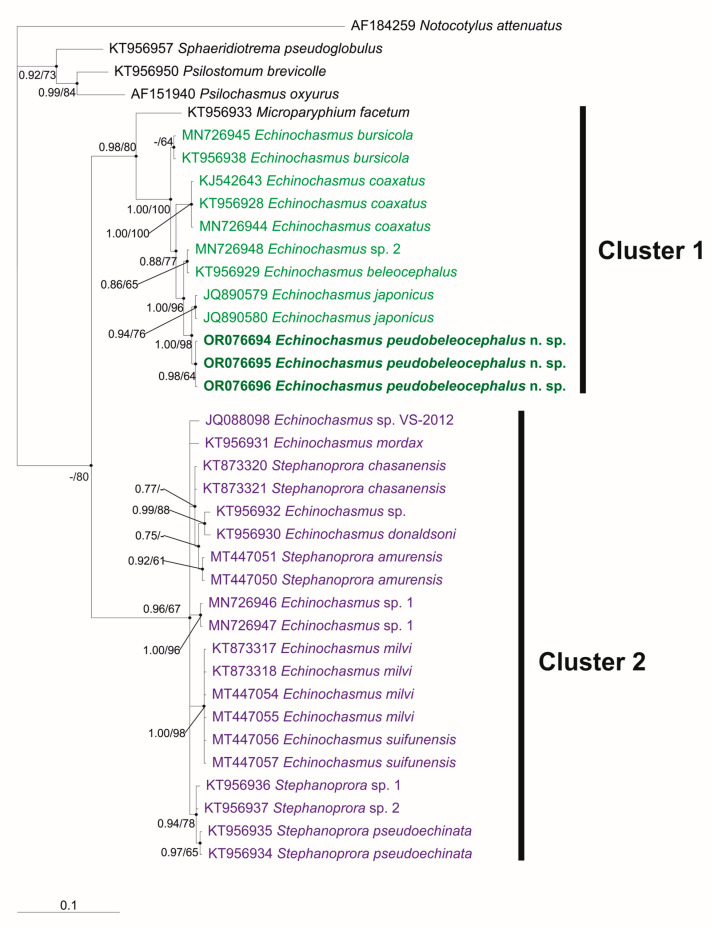
The phylogeny of the family Echinochasmidae based on the 28S rRNA gene sequences (1020 bp) using the Bayesian Inference (BI) algorithm. Values of a posterior probability ≥ 60 for BI and nodal support of ≥0.60 for Maximum Likelihood (ML) are shown at the nodes, respectively. The scale bar indicates the number of substitutions per site. The sequences obtained in the present study are highlighted in bold. The outgroup species are listed in Table 2.

The reconstruction of phylogenetic relationships among Echinochasmidae using *28S* sequences showed a subdivision of members of this family into two clusters with an intergeneric distance between them being 6.3% (without a member of the genus *Microparyphium*, removed from the analysis (Figure 2)). The level of genetic distances between *Microparyphium facetum* and representatives of *Echinochasmus* from Cluster 1 were in a range from 5 to 6%. The distances between *Echinochasmus* species grouped into Cluster 1 ranged from 0.3 to 2%. In Cluster 2, where members of the family attributed to *Echinochasmus* were grouped with *Stephanoprora*, the differences ranged from 0.2 to 1.9% (Table 3).

In Cluster 1, the individuals obtained in this study were grouped with four other species of *Echinochasmus* and a representative of *Microparyphium*. However, they formed a single branch with *E. japonicus* from Vietnam with a genetic distance between them being 0.3%, which was minimal within the Cluster. The European *E. beleocephalus* and *Echinochasmus* sp. 1, not identified as species, formed a separate branch that occupied an external position relative to our specimens and *E. japonicus* from Vietnam with 0.7% differences from these two species. The rest of the species from Cluster 1 held an external position relative to all the above-mentioned representatives of *Echinochasmus* with a range of distances from 0.7 to 2%. Judging by the values of interspecies differences within the family Echinochasmidae, previously estimated by analyses of *28S* in the studies of Schwelm et al. (2020, 0.2–1.5%) [16] and Tatonova et al. (2020, 0.2–2.6%) [7], the specimens of *E. japonicus* from Vietnam and those in our material were representatives of a different *Echinochasmus* species. Therefore, we attributed them to a new species, *Echinochasmus pseudobeleocephalus* n. sp.

## 5. Discussion

The results of the phylogenetic reconstruction of relationships in the family Echinochasmidae including new data for representatives of *Echinochasmus* were consistent with those presented in the publications by Tkach et al. (2016) [5] and Tatonova et al. (2020) [7] and confirmed the identification of the clusters that comprised representatives of *Echinochasmus* and *Stephanoprora* with similar numbers of head-collar spines and morphologies of cercariae. However, as in the studies by Tkach et al. (2016) [5] and Tatonova et al. (2020) [7], it was found that the genetic distances within the clusters in the reconstruction based on *28S* were in the range of intrageneric level, while the difference between the clusters reached the intergeneric level. In Cluster 1, which included the *Echinochasmus* species, the adoral disk of mature individuals was equipped with 24 head-collar spines, and cercariae had short tails, equal to or slightly longer than the body length. The species affiliation of one of the specimens included in this cluster, identified as *Echinochasmus bursicola*, remains unresolved. The trematodes with 20, 22, and 24 head-collar spines were described as this species [1,23,27,28,29,30]. Tkach et al. (2016) [5] obtained molecular data for individuals with 24 spines from Ukraine and attributed them to *E. bursicola*. Nucleotide sequences of *28S* identical to those obtained by Tkach et al. (2016) [5] were also found in short-tailed cercariae of *Echinochasmus* from snails *Bithynia tentaculata* collected in Germany [16]. On the basis of this marker, the trematodes found in Germany at the cercarial stage were also attributed to *E. bursicola*. The vast majority of studies on echinochasmids showed that different species are characterized by a different number of head-collar spines. If we assume that the adult trematodes in the material of Tkach et al. (2016) [5] were, indeed, *E. bursicola*, then the rest of the individuals with 20 and 22 spines should belong to other species. This also applies to the Russian Far Eastern trematodes identified as *E. japonicus* but having 22 head-collar spines, in contrast to the individuals of this species with 24 spines described by Tanabe (1926) [31]. As for *28S*, this marker does not always provide reliable species differentiation for echinochasmids. There is a report about the identity of this gene sequence between specimens belonging to different Echinochasmidae species [7]. In view of the considerations above, the question as to which individuals belong to *E. bursicola* and which do not, in our opinion, still remains open.

Cluster 2 includes representatives of *Echinochasmus* and *Stephanoprora* with 20–22 head-collar spines and long-tailed cercariae. In this group of echinochasmids, seven species were identified. However, only for five of them, the determination of species affiliation was based on both the morphology of developmental stages and nucleotide sequences, which were obtained in the same studies. The set of such data provides sufficient accuracy in the attribution of genetic characteristics to the species identified. Unfortunately, for a number of specimens in Cluster 2, including those with identified species, there were no morphological data. This greatly complicates the resolution of their taxonomy and the determination of the level of relationships with other individuals of Echinochasmidae. For example, the morphology and photographs of the cercariae of *Echinochasmus* sp. 1 (MN726946 and MN726947) [16] indicate their similarity with cercariae of *Stephanoprora*, to which they may probably belong. Furthermore, there is still no complete clarity as regards the trematodes identified as *E. donaldsoni* Beaver, 1941, for which partial *28S* have been sequenced but no morphological characteristics have been provided for the studied individuals. If these specimens actually belong to *E. donaldsoni*, then the question remains as to whether the morphology of their cercariae matches that of the specimens grouped in Cluster 2. Thus, according to Beaver (1941) [32], the tail length in cercariae of *E. donaldsoni* is equivalent to the body length, while Yamaguti (1975) [22] described the tail of *E. donaldsoni* cercariae as “tail powerful with annular ridges throughout its length when contracted”, which is typical of *Stephanoprora* cercariae. However, judging by the sizes that the author reported [22] for these cercariae, the lengths of their tails are equivalent to the body length. Therefore, in our opinion, additional information including both morphological and molecular data is required to clarify the taxonomic affiliation of the specimen identified as *E. donaldsoni* (KT956930).

It should also be noted that the principle of differentiation between Echinochasmidae on the basis of morphological criteria is also followed in reconstructions using other nuclear markers [7]. Moreover, if relationships between individuals of the family in the clusters based on *28S* have an intrageneric level, and an intergeneric level is observed between individuals of different clusters, then echinochasmids are divided into groups of a higher taxonomic rank on the basis of another nuclear marker, ITS2 rDNA region [7]. However, in view of the limited amount of morphological and molecular data available to date for members of Echinochasmidae, claiming these taxonomic problems as resolved is premature. The taxonomy of this large group of trematodes will only be reliable if both morphological and molecular studies cover a sufficiently large number of species of this family.

## 6. Conclusions

In this study, a life cycle experiment was set up and the morphology of the developmental stages of an allegedly new trematode species from the Russian Far East was examined. As the new molecular data have shown, the obtained individuals from the genus *Echinochasmus* represent a new species despite its morphological characteristics similar to those of the European *E. beleocephalus*. Thus, the Far Eastern trematode has been given a new scientific name, *Echinochasmus pseudobeleocephalus*. Apparently, *Echinochasmus pseudobeleocephalus* n. sp. and *E. japonicus* and *E. beleocephalus* from different regions are a group of cryptic species whose successful differentiation requires a combination of data on the morphology of developmental stages and the molecular characteristics of individual species obtained in a single study.

## Figures and Tables

**Table 1:** List of species used in the analysis with sequences of the 28S rRNA gene and other associated information.

Species	Developmental Stage	Accession Numbers (GenBank)	Reference	Country	Sequence Length, bp
family Echinochasmidae					
*Echinochasmus pseudobeleocephalus* n. sp.	Adult	OR076694, OR076695	this study	Russia	701
	Cercaria	OR076696			
*Echinochasmus japonicus*	Adult	JQ890579, JQ890580	[15]	Russia	1370
*Echinochasmus beleocephalus*	Adult	KT956929	[5]	Ukraine	1178
*Echinochasmus* sp. 2	Cercaria	MN726948	[16]	Germany	1199
*Echinochasmus coaxatus*	Adult	KJ542643	[17]	Ukraine;	969
	Adult	KT956928	[5]	Ukraine	1200
	Cercaria	MN726944	[16]	Germany	1200
*Echinochasmus bursicola*	Adult	KT956938	[5]	Ukraine	1173
*Microparyphium facetum*	Adult	KT956933	[5]	USA	1291
*Echinochasmus* sp. VS-2012	Cercaria	JQ088098	[17]	Lithuania	1258
*Echinochasmus mordax*	Adult	KT956931	[5]	Ukraine	1175
*Stephanoprora chasanensis*	Adult	KT873320, KT873321	[6]	Russia	1369
*Echinochasmus* sp.	Adult	KT956932	[5]	USA	1161
*Echinochasmus donaldsoni*	Adult	KT956930	[5]	USA	1240
*Stephanoprora amurensis*	Adult	MT447050, MT447051	[7]	Russia	1145
*Echinochasmus* sp. 1	Cercaria	MN726946, MN726947	[16]	Germany	1199
*Echinochasmus milvi*	Adult	KT873317, KT873318	[6]	Russia Russia	1369
		MT447054, MT447055	[7]		1145
*Echinochasmus suifunensis*	Adult	MT447056, MT447057	[7]	Russia	1145
*Stephanoprora* sp. 1	Adult	KT956936	[5]	USA	1181
*Stephanoprora* sp. 2		KT956937			
*Stephanoprora pseudoechinata*	Adult	KT956934, KT956935	[5]	Ukraine	1249
Outgroup					
*Psilostomum brevicolle*	Adult	KT956950	[5]	Ukraine	1292
*Psilochasmus oxyurus*	Adult	AF151940	[18]	Ukraine	1239
*Sphaeridiotrema pseudoglobulus*	Adult	KT956957	[5]	USA	1206
*Notocotylus attenuatus*	Adult	AF184259	[19]	Ukraine	1261

**Table 2:** Morphometric parameters of adult *Echinochasmus* individuals (µm).

Characters	*E. pseudobeleocephalus* n. sp.			*E. japonicus*				*E. beleocephalus*	
	This Study			[22]	[3]	[4]	[15]	[23]	[4]
	Holotype	Range (*n* = 7)	Mean						
Body length (Bl)	454	454–554	502	600–900	540–620	780–810	520–580	715–924	550–620
Body width (Bw)	173	139–173	152	160–180	150–180	220–250	162–235	253–330	130–170
Bw/Bl (%) *	38.1	27.2–38.1	30.3						
Forebody length (Fo)	204	204–258	234						
Fo/Bl (%) **	44.9	44.4–48.6	46.6						
Oral sucker length	39	31–39	36	38–42	40–51	45–50	23–35	47–51	34–39
Oral sucker width	35	35–39	37	38–42	40–57	56	27–42	47–51	39–42
Ventral sucker length	69	62–81	70	70–96	68–91	95–110	50–65	132–143	59–70
Ventral sucker width	73	65–85	72	70–96	74–86	67–89	54–77	132–154	62–73
Ratio of suckers’ lengths	1.78	1.59–2.10	1.94						
Ratio of suckers’ widths	2.09	1.67–2.09	1.95						
Head-collar width	92	85–116	96						
Prepharynx length	35	31–50	40	30–60	46–68	28–34	15–42	33–38	48–50
Pharynx length	35	35–42	38	35–39	34–46	67–84	19–27	51–56	28–40
Pharynx width	39	31–39	34	27–32	34–51	45–50	23–31	51–56	34–39
Oesophagus length	58	58–100	78	110–210	97–120	95–130	92–96	132–198	67–130
Ovary length	35–39	31–39	34	36–48	40–51	41–60	35–42	38–43	34–42
Ovary width	31–35	27–35	32	22–30	46–67	38–49	35–42	47	31–50
Anterior testis length	42	35–62	48	60–75	40–68	83–135	58–80	43–88	50–70
Anterior testis width	58	53–77	61	54–80	34–46	100–132	65–92	34–88	59–80
Posterior testis length	42	39–62	47						
Posterior testis width	58	46–62	59						
Cirrus sac length	58	58–69	61	75–90	–	91–130	50–65	86–132	62–81
Cirrus sac width	46	39–46	41	36–48	–	60–74	42–58	66–77	34–48
Post-testicular field length	69	58–69	65						
Egg length		deformed		77–90	63–86	84–89	80	73–81	84
Egg width				51–57	46–57	50	53	34–43	61
Number of head-collar spines	24	24	24	24	24	22	24	24	24

* Bw/Bl, body width as percentage of body length; ** Fo/Bl, forebody length as percentage of body length.

**Table 3:** Genetic distances (below the diagonal) and standard error estimate (above the diagonal) between species of family Echinochasmidae e based on the 28S rRNA gene sequences.

	No	Species	1	2	3	4	5	6	7	8	9	10	11	12	13	14	15	16	17	18
Cluster 1 *	1	*Microparyphium facetum*		0.008	0.008	0.009	0.009	0.009	0.009	0.008	0.007	0.008	0.007	0.008	0.008	0.007	0.008	0.008	0.007	0.008
	2	*Echinochasmus bursicola*	0.047		0.004	0.003	0.003	0.004	0.004	0.009	0.009	0.009	0.009	0.009	0.009	0.009	0.009	0.009	0.008	0.008
	3	*E. coaxatus*	0.059	0.011		0.004	0.004	0.005	0.005	0.010	0.009	0.009	0.010	0.010	0.010	0.010	0.010	0.010	0.009	0.009
	4	*Echinochasmus.* sp. 2	0.054	0.007	0.016		0.000	0.003	0.003	0.010	0.009	0.009	0.010	0.010	0.010	0.009	0.010	0.010	0.009	0.009
	5	*E. beleocephalus*	0.054	0.007	0.016	0.000		0.003	0.003	0.010	0.009	0.009	0.010	0.010	0.010	0.009	0.010	0.010	0.009	0.009
	6	*E. japonicus*	0.059	0.011	0.020	0.007	0.007		0.002	0.009	0.009	0.009	0.009	0.009	0.009	0.009	0.010	0.010	0.009	0.009
	7	*E. peudobeleocephalus*	0.059	0.011	0.020	0.007	0.007	0.003		0.009	0.009	0.009	0.009	0.009	0.009	0.010	0.010	0.010	0.009	0.009
Cluster 2 *	8	*Echinochasmus* sp. VS-2012	0.062	0.059	0.069	0.066	0.066	0.059	0.062		0.004	0.003	0.004	0.004	0.004	0.004	0.005	0.005	0.003	0.003
	9	*E. mordax*	0.059	0.056	0.066	0.063	0.063	0.056	0.059	0.011		0.003	0.004	0.004	0.003	0.005	0.005	0.005	0.004	0.004
	10	*Stephanoprora chasanensis*	0.060	0.054	0.064	0.062	0.062	0.054	0.057	0.007	0.007		0.003	0.003	0.002	0.004	0.004	0.004	0.003	0.003
	11	*Echinochasmus* sp.	0.067	0.062	0.072	0.069	0.069	0.062	0.064	0.011	0.011	0.007		0.003	0.003	0.005	0.005	0.005	0.004	0.004
	12	*E. donaldsoni*	0.064	0.059	0.069	0.066	0.066	0.059	0.062	0.011	0.011	0.007	0.006		0.003	0.005	0.005	0.005	0.004	0.004
	13	*S. amurensis*	0.060	0.057	0.067	0.064	0.064	0.057	0.060	0.010	0.007	0.003	0.007	0.007		0.004	0.004	0.004	0.003	0.003
	14	*Echinochasmus* sp. 1	0.059	0.056	0.066	0.063	0.063	0.059	0.062	0.014	0.014	0.010	0.017	0.017	0.013		0.005	0.005	0.004	0.004
	15	*E. milvi*	0.067	0.062	0.072	0.063	0.063	0.062	0.064	0.014	0.017	0.010	0.017	0.017	0.013	0.017		0.000	0.004	0.004
	16	*E. suifunensis*	0.067	0.062	0.072	0.063	0.063	0.062	0.064	0.014	0.017	0.010	0.017	0.017	0.013	0.017	0.000		0.004	0.004
	17	*Stephanoprora* sp.	0.054	0.054	0.064	0.062	0.062	0.057	0.060	0.007	0.010	0.006	0.013	0.013	0.006	0.010	0.013	0.013		0.002
	18	*S. pseudoechinata*	0.056	0.056	0.066	0.063	0.063	0.059	0.062	0.009	0.011	0.007	0.014	0.014	0.007	0.011	0.014	0.014	0.001	

* Representatives of Clusters 1 and 2 and the distances within the clusters are indicated in green and violet, respectively.

## Data Availability

The obtained sequences of the 28S gene for the new species were deposited in the National Center for Biotechnology Information database (NCBI, https://www.ncbi.nlm.nih.gov, accessed on 18 August 2023).

## Supplementary Materials

The following supporting information can be downloaded at: https://www.mdpi.com/article/10.3390/ani13203236/s1, Figure S1. Sampling location (the Arsenyevka river) of *Echinochasmus pseudobeleocephalus* n.sp. in this study.
