# Supplementary material for: A Description of Echinochasmus pseudobeleocephalus n. sp. (Echinochasmidae) Based on Morphological and Molecular Data
Source: Animals (Basel). 2023 Oct 17;13(20):3236. doi: 10.3390/ani13203236 (PMC10603625; doi:10.3390/ani13203236)
Supplement: Supplementary file 1 [file animals-13-03236-s001.zip › animals-2592048-supplementary.pdf]

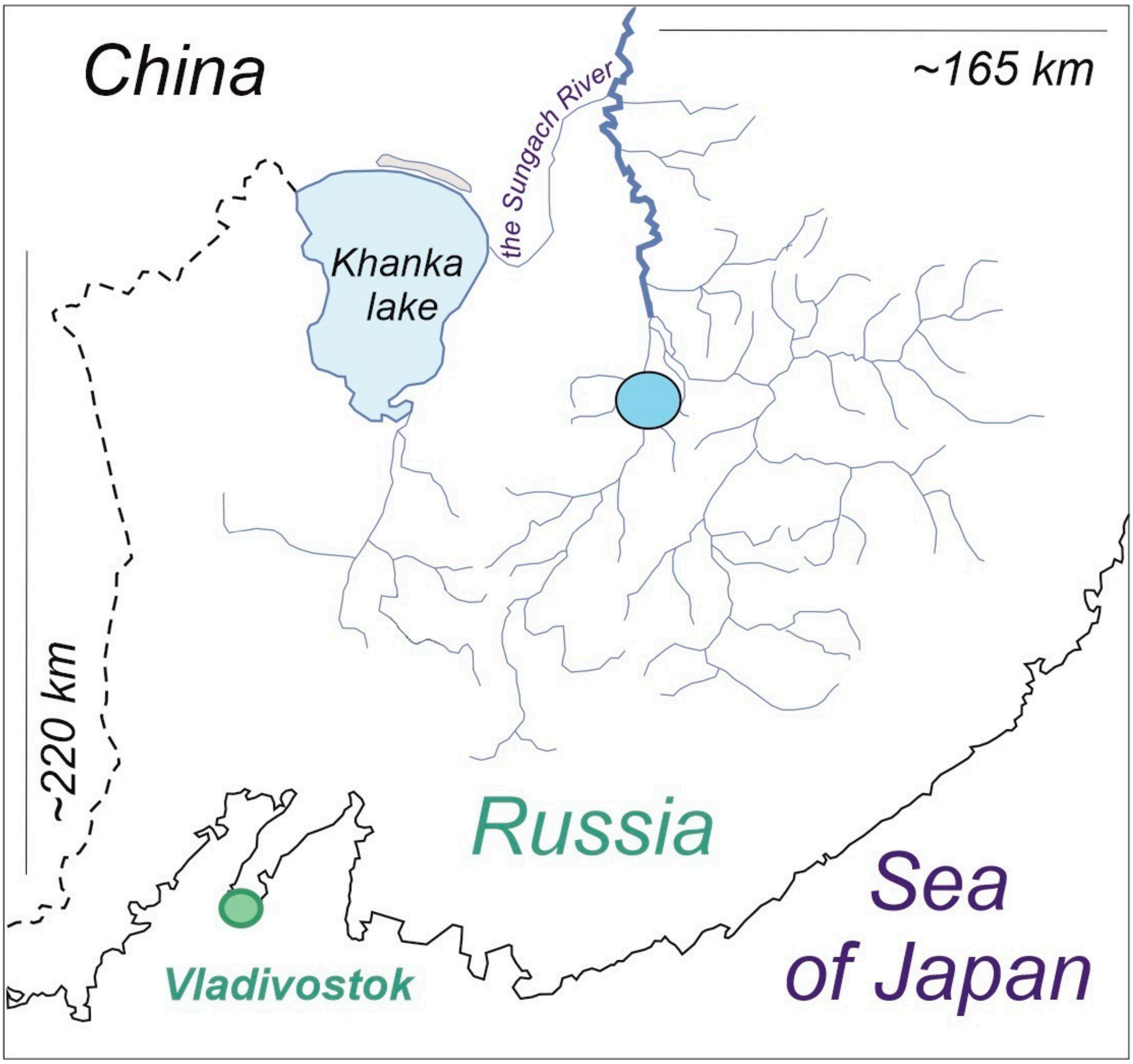

Figure S1. Sampling location (the Arsenyevka river) of *Echinochasmus pseudobeleocephalus* n.sp. in this study.
